# Personality Disorder and Crisis in the COVID-19 Pandemic

**DOI:** 10.1192/bjo.2024.243

**Published:** 2024-08-01

**Authors:** Amar Sethi, Andrew Howe

**Affiliations:** 1 East and North Hertfordshire NHS Trust, Stevenage, United Kingdom; 2 South London and Maudsley NHS Foundation Trust, London, United Kingdom

## Abstract

**Aims:**

To identify if COVID-19 has changed the experience for patients under the care of Crisis Resolution Home Treatment teams (HTT).

To identify if COVID-19 altered the response for HTT patients in the context of Personality Disorder (PD).

To provide useful demographic and experiential information about patients using HTT with PD during crisis.

**Methods:**

Data regarding the demographics of patients with personality disorders under the care of the Croydon crisis home treatment team were collected retrospectively for two, predetermined time windows. The first window was pre-COVID-19 (26/03/2019–25/03/2020) and the second window was during COVID-19 (26/03/2020–25/03/2021). The demographics of patients with personality disorder referred to the team during these two time periods included were compared.

**Results:**

More patients with personality disorder were referred to the Croydon HTT during COVID-19 (n = 82) when compared with the window before (n = 58). The proportion of referred patients with Emotionally Unstable Personality Disorder (EUPD) was constant before and during COVID-19. The average length of stay reduced from 22.6 days before COVID-19 to 18.7 days during COVID-19. The proportion of rejected referrals to the HTT of patients with personality disorder increased during COVID-19. Finally, the proportion of BAME (Black, Asian, Minority Ethnicity) referrals of patients with personality disorder increased during COVID when compared with before, with this finding not being replicated in any other ethnic group.

**Conclusion:**

Increased numbers of referrals may indicate worsening mental health in the community. This may have been compounded by an inability of community mental health teams and inpatient services to meet such an increase in demand for services. An overall reduction in inpatient admissions during COVID-19 supports this idea. There was a relatively larger drop in duration of admission for patient with personality disorder during COVID-19, when compared with all patients. This may be due to staff feeling unable to offer quick management for patients with personality disorder. Subsequently, staff may have selectively discharged such patients earlier. The rate of rejected referrals to the Croydon HTT was consistently higher than the acceptances both pre- and post- COVID-19. Therefore, HTT clinicians may feel unable to adequately treat PD.

In conclusion, the number of referrals to the HTT increased during COVID-19, however, with a reduced average duration of stay with HTT for patients. The rejection rates for personality disorder patients were consistently higher than for other patient groups, both before and during COVID-19. Additionally, the proportion of patients with personality disorder from BAME backgrounds increased during COVID-19.

